# Public Policy Should Foster Alzheimer’s Treatment Availability: Comment on the Draft US Medicare Decision to Limit Payment for Aducanumab (Aduhelm^™^) to Patients Participating in Clinical Trials

**DOI:** 10.14283/jpad.2022.25

**Published:** 2022

**Authors:** J. Cummings

**Affiliations:** Chambers-Grundy Center for Transformative Neuroscience, Department of Brain Health, School of Integrated Health Sciences, University of Nevada Las Vegas (UNLV), Las Vegas, NV, USA

Aducanumab (Aduhelm^™^) was approved by the United States (US) Food and Drug Administration (FDA) for the treatment of Alzheimer’s disease on June 7, 2021. Soon after, the approved labeling was adjusted to direct treatment to mild cognitive impairment (MCI) due to Alzheimer’s disease (AD) and mild AD dementia reflecting the severity of cognitive impairment among participants in the clinical trials that led to the approval (1, 2).

Individuals over age 65 in the US are entitled to have the cost of prescription drugs partially paid by the Center for Medicare and Medicaid Services (CMS; “Medicare”) if they participate in an approved insurance plan. Most people pay a monthly fee for this benefit. When a new agent becomes available it may be subject to a National Coverage Determination (NCD) to ascertain if CMS will pay for the drug and under what circumstances. CMS covers drugs that are considered “reasonable and necessary” for the treatment of an illness. The covered treatment must be shown to meaningfully improve health outcomes. The NCD process can lead to approval of coverage, denial for coverage, or limited coverage (3).

On January 11, 2022, Medicare Issued its draft NCD on Coverage with Evidence Development (CED) for aducanumab which limits coverage to patients participating in CMS-approved randomized controlled clinical trials supported by the US National Institutes of Health (NIH) (3). This decision, if sustained, would greatly limit and delay availability of aducanumab to patients who could benefit from treatment. This decision has many foreseeable adverse consequences. The draft proposal must be understood in the context of the available efficacy data and the process of accelerated approval. These are discussed here followed by a description of the CMS proposal to limit the coverage of aducanumab to participants in trials. The proposed CED is not in the best interest of patients with early AD or the field of AD treatment development. The current decision is a draft of the proposed CED and is subject to change until a final decision is rendered on April 11th, 2022. Public comment on the decision is invited until February 10, 2022, and there is an opportunity to modify this draft determination (https://www.cms.gov/medicare-coverage-database/search.aspx).

## Aducanumab

### Background

**Figure 1.**
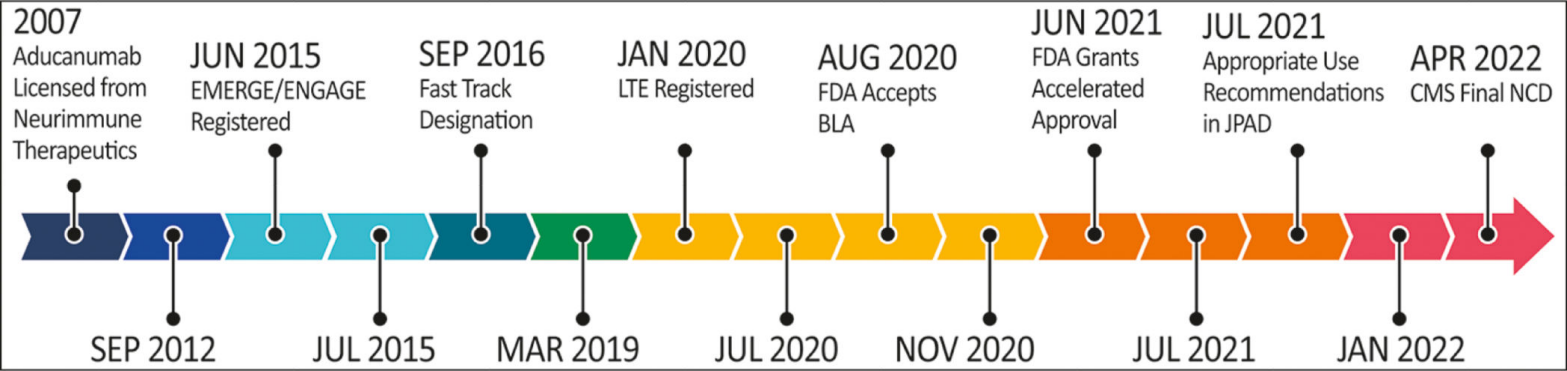
History of aducanumab from laboratory discovery through clinical trials, regulatory review, and CMS coverage decisions Trial dates are those derived from clinicaltrials.gov. BLA – Biologics License Application; CED – Coverage with Evidence Determination; CMS – Center for Medicare and Medicaid Services; FDA – Food and Drug Administration; LTE – Long Term Extension; NCD – National Coverage Determination (illustrator M de la Flor, PhD;).

Aducanumab is an anti-amyloid IgG1 monoclonal antibody (mAb) directed to the N-terminus of the amyloid beta peptide (Aß) occurring in aggregates (5). Nonclinical studies showed that antibody administration led to marked lowering of brain Aß plaques in animal models of AD (4). Figure 1 summarizes the history of the development of aducanumab.

### Efficacy

The first clinical trial of aducanumab was the PRIME study, a Phase 1B dose-finding trial (5). Participants shown to have brain amyloid consistent with AD using amyloid positron emission tomography (PET) received 1, 3, 6, or 10 mg/kg for 12 months. Time- and dose-dependent reduction of brain amyloid was observed on amyloid PET in the 3, 6, and 10 mg/kg dose groups. Patients in the 6 and 10 mg dose cohorts reached brain amyloid levels near those considered to be normal at the end of the trial. The study was not powered for clinical outcomes. The Clinical Dementia Rating – Sum of Boxes (CDR-SB) and the Mini Mental State Examination (MMSE) showed nominally significantly less decline in the 10 mg/kg dose group than the placebo group; the Neuropsychological Test Battery and Free and Cued Selective Reminding Test showed no drug-placebo difference. A correlation was observed between amyloid lowering as measured by the amyloid PET composite standardized update value ratio (SUVR) and the CDR-SB.

On the basis of the PRIME observations, two identical Phase 3 trials --- ENGAGE and EMERGE --- with 1643 planned participants each were launched (6). The results of these studies have not been published and the data discussed here are derived from the comprehensive presentations of information to the FDA (8). Participants had early AD confirmed by amyloid PET and had MMSE scores of 24–30. ENGAGE was launched and began participant recruitment before EMERGE. Patients were randomized to a high dose, low dose, or placebo. Low dose was 3 mg/kg for apolipoprotein E ε4 (APOE4) gene carriers and 6 mg/kg for APOE4 noncarriers based on knowledge of the increased risk of amyloid related imaging abnormalities (ARIA) in those with an APOE4 gene. High dose was 10 mg/kg for participants without an APOE4 gene and 6 mg/kg for those who had the APOE4 gene. After trial initiation, a protocol amendment allowed the high dose to be increased to 10 mg/kg in the APOE4 gene carriers. A futility analysis of pooled data from the two trials conducted when half of the patients had received 18 months of treatment indicated that the conditional probability of success was low, and the trials were terminated. The final analyses of the blinded data of the intent-to-treat population revealed that the high dose arm of the EMERGE trial met its prespecified primary outcome (CDR-SB) and ENGAGE did not. All prespecified secondary outcomes of EMERGE showed a significant drug-placebo difference. Compared to placebo, the high dose treatment was associated with 22% slowing of decline on the CDR-SB, 18% on the MMSE, 27% on the Alzheimer’s Disease Assessment Scale – cognitive subscale (ADAS-cog), and 40% on the Alzheimer’s Disease Cooperative Study Activities of Daily Living Mild Cognitive Impairment version (ADCS-ADL-MCI). An exploratory assessment of behavioral changes using the Neuropsychiatric Inventory (NPI) showed an 87% drug-placebo difference in favor of aducanumab. There was significant dose-dependent lowering of brain amyloid evident at week 26 which was more marked at week 78. Significant reductions in cerebrospinal fluid (CSF) phospho-tau (p-tau) and total tau (t-tau) were observed in participants of the CSF substudy (N = 78). Slowing of decline on the CDR-SB was correlated with reduction of brain amyloid. Primary and secondary outcomes of the ENGAGE study showed no drug-placebo differences. Amyloid reduction in ENGAGE was somewhat less than observed in EMERGE. There was a lower cumulative exposure to aducanumab in the ENGAGE study and fewer patients who received the high dose of 10 mg/kg for the planned period of treatment (22% in ENGAGE, 29% in EMERGE). These dose discrepancies may partially explain the difference in outcomes of the EMERGE and ENGAGE trials. Patients who had at least eight consecutive doses of 10 mg/kg in ENGAGE had a similar slowing of decline to that observed in EMERGE.

The 22% slowing on the primary outcome has been challenged as inappropriately small to warrant the treatment. Slowing by this amount translates to approximately one additional year in the typical 5-year period in the MCI stage of AD prior to entering the terminal dementia phase of the illness; patients should be empowered to decide if this degree of slowing is desirable for them.

### Safety

ARIA of the effusion type (ARIA-E) and of the hemorrhagic type (ARIA-H) were observed in trials of aducanumab (6, 7). In pooled EMERGE and ENGAGE studies, ARIA-E was observed in 35% of patients receiving high dose aducanumab; 14.5% had severe ARIA-E and 6.1% discontinued trial participation because of ARIA-E. The frequency of ARIA-E was higher in those with at least one copy of the APOE4 gene (43%). Among participants with high dose exposure, 19.1% had ARIA-H, and 14.6% had superficial siderosis consistent with hemosiderin deposits resulting from hemorrhage into the brain parenchyma or on the pial surface. Most ARIA-E (74%) produced no symptoms.

### Summary

The irregular aspects of the trials of aducanumab with premature termination suggest caution in interpreting the results. The consistent pharmacodynamic relationships among dose, duration, amyloid lowering, and slowing of clinical decline (as seen on the CDR-SB) support the efficacy of aducanumab. The effect on “downstream” biomarkers (p-tau, t-tau) associated with cognitive decline in many studies is additional evidence of the impact of aducanumab on the biology of AD and disease modification (8). An efficacy conclusion for anti-amyloid mAbs is consistent with observations emerging from studies of other agents in this class (discussed below).

## Accelerated Approval

Aducanumab was approved by the FDA using the regulatory mechanism of accelerated approval. This approval pathway was used for 14 of 50 (28%) approvals by the FDA in 2021 and is commonly used for cancer therapies (9). Accelerated approval is employed when a potential treatment for a life-threatening illness has an effect on a surrogate endpoint that is reasonably likely to predict clinical benefit (10). Post-marketing confirmatory trials may be required to verify the anticipated effect. Uncertainty about whether clinical benefit will be verified, and the possibility of undiscovered risks are the primary reasons that accelerated approval is reserved for drugs intended to treat serious conditions. As described by the FDA, determining whether an endpoint is reasonably likely to predict clinical benefit is a matter of judgment that will depend on the biological plausibility of the relationship between the disease and the endpoint and the empirical evidence supporting that relationship. The FDA guidance on accelerated approval notes that this regulatory mechanism is consistent with the agency’s commitment to flexibility regarding the evidence required to support product approval for the treatment of serious or life-threatening diseases with limited therapeutic options (10). There are provisions for withdrawal of approval if post-approval trials fail to verify the predicted clinical advantages. A confirmatory trial of aducanumab is required and approval can be retracted if efficacy is not supported.

Reduction of amyloid plaques as shown on amyloid PET is the biomarker on which accelerated approval was based. Plaque reduction is not a fully validated surrogate and there is a lack of consensus on the predictive value of plaque removal. There have been many failures of anti-amyloid therapies suggesting that approval based on anti-amyloid effects does not have an adequate scientific rationale (11). Nearly all the failed programs have been directed at pre-plaque species of Aβ and did not decrease amyloid plaques. Plaques have been linked by many observations to cognitive impairment in AD, and the effect of mAbs on plaques meets the standard of “reasonably likely” to predict clinical benefit (8, 12). Two other sponsors of mAb development programs are pursuing accelerated approval based on plaque lowering effects (Eisai for lecanemab and Eli Lilly for donanemab); both mAbs have shown preliminary evidence of slowing of clinical decline in addition to the biomarker efficacy (13, 14). To what extent plaque removal is the critical biology of the therapeutic response or is a marker for an effect on toxic amyloid oligomers, tau species, or inflammation remains to be determined. Biomarkers linked to effects on these non-amyloid aspects of AD biology might eventually be shown to be reasonably likely to predict clinical benefit and be employed in future accelerated approval strategies.

Accelerated approval is intended to make drugs available to patients with life-threatening diseases such as AD while additional evidence to confirm efficacy and safety is generated. The decision of CMS to limit aducanumab to clinical trials is at variance with the purpose of this approach and inconsistent with the intent of the FDA to provide a mechanism for accelerated access to aducanumab for appropriate patients.

## CMS Draft Decision

In their draft determination, CMS proposed to cover FDA approved mAbs directed against plaque amyloid for the treatment of AD under a Coverage with Evidence Development (CED) approach limiting coverage of mAbs to patients participating in CMS-approved randomized controlled trials supported by the NIH (3). All trials must be conducted in a hospital-based outpatient setting. Based on the lack of inclusion of underserved populations in past trials, CMS requires enrollment of a patient population representative of those diagnosed with AD. The CMS draft proposal addresses anti-amyloid mAbs as a class.

This approach is not patient-centered and will greatly delay access to treatment for individuals with early AD. This proposed determination is predicted to reduce interest in treatment development when the increasing population of those with and at risk for AD requires innovative solutions for their cognitive and functional impairment.

### Controlled Clinical Trials

Requiring that aducanumab be studied in clinical trials conducted in a hospital setting greatly limits the number of patients with early AD who could receive treatment with coverage. Proposed trials are likely to be placebo-controlled (not explicitly required in the proposed decision memo but implied by the trial requirements). In placebo-controlled trials, patients seeking treatment must agree to be randomized to drug or placebo to have a chance of receiving therapy. Severely limiting access to an approved therapy in this way is coercive.

Requiring that the trial be funded by NIH attaches additional limits to the availability of treatment. NIH funding is directed mostly to trial sites associated with academic medical centers restricting access to therapy to those living in nearby areas. Academic trials sites are rarely in minority neighborhoods and are not situated to allow recruitment of rural populations (also under-represented in clinical trials). NIH funding for trials is less than that invested by pharmaceutical companies and recruiting an adequate number of participants to adequately power a trial to demonstrate benefit may be difficult if not impossible. Funding by NIH is subject to extensive peer review on scheduled review cycles. Trials as proposed by CMS would require 1–2 years to secure funding plus 3–5 years to conduct and analyze, delaying the availability of an approved treatment that could be available NOW. NIH funding has been instrumental in supporting Phase 2 proof-of-concept and dose-finding studies; it has rarely been used for trials of the type proposed by CMS that more closely resemble Phase 3 trials (15).

CMS requires that approved trials must determine if treatment with an anti-amyloid mAb results in a statistically significant and meaningful reduction in decline in cognition and function. CMS chose the CDR-SB to exemplify what constitutes a clinically meaningful improvement as a primary outcome. To define minimal clinically important differences they suggest a 1–2 point increase in CDR-SB, plus a 1–3 point decrease in MMSE and 3–5 point increase in Functional Activities Questionnaire (FAQ) (16). The population from which these figures were derived is a biologically unconfirmed non-trial population with data contributed to the US National Alzheimer Coordinating Center; these patients differ substantially from those to be included in CMS-approved trials of patients with early AD confirmed by positive amyloid imaging and healthy enough to participate in a trial. No means of translating outcomes on these tools to the stated purpose of the trials to demonstrate “meaningful improvement in health outcomes” is provided.

The CMS proposal does not state how many trials with meaningful results are required to support coverage although the proposed decision memo refers to “trials.” The expected relationship of results from different trials or the required consistency of outcomes is not discussed.

Noting the lack of diversity in previous trials, the higher prevalence of AD in Black and non-White Americans, and the directives in Executive Order 13985, Advancing Racial Equity and Support for Underserved Communities through the Federal Government, CMS proposes requiring that the patients included in each trial be representative of the national population diagnosed with AD. This is a noble aspiration. It is also an unrealistic expectation given the known challenges in recruiting underrepresented and underserved populations to trials. We must improve inclusion of diverse populations in trials; this is a long-term goal requiring trial infrastructure not currently available and trust that has not been built. Requiring a representative sample in the CMS trial will delay the trial and limit the availability of drug treatment to both minority and majority culture patients. The mandate ignores other underserved populations such as those dwelling in rural areas who have essentially no opportunity to reach trial sites and treatment in a trial.

Aducanumab is approved and can be purchased through self-pay mechanisms independent of coverage by CMS. Limitation of treatment coverage to those in trials means that persons in limited financial circumstances will have limited access to therapy while those with financial means will have access to treatment. CMS requirements will increase the lack of equity in AD care in the US.

Unlike participating in an industry-sponsored trial where experimental therapy is provided without cost, CMS typically does not cover the full costs of drugs and a substantial co-payment may be leveraged for aducanumab trial participants. Up to 50 percent of patients in a trial (depending on the use of a placebo and the randomization ratio) may be paying for placebo. These circumstances will further disincentivize trial participation.

Together these observations argue against the requirement for a CED comprised of clinical trials to provide coverage for mAbs.

### Real World Use

CMS expresses concerns about harms to patients that would be treated outside the context of the safety monitoring of a controlled trial. Clinicians agree that the occurrence of ARIA can have serious consequences and must be monitored. In the EMERGE and ENGAGE trials, most ARIA events (72.3%) occurred within the period of the first eight doses (7 months) and vigilance early in the course of therapy is key to safe introduction of aducanumab. Seventy-four percent of those with MRI-proven ARIA had no symptoms. Of the 26 percent who exhibited symptoms, most had headache with fewer having confusion, dizziness, or nausea (7). A few patients have severe symptoms including seizures, and one death has occurred a patient with a complex medical history and ARIA. The risks of ARIA do not exceed those of cancer therapies that are routinely covered by CMS; limitation of coverage for treatment of patients with AD is disease-based discrimination.

To assist clinicians in real world settings with the management of ARIA, an Expert Panel developed Appropriate Use Recommendations that describe the baseline magnetic resonance imaging (MRI) findings suggesting the patient may not be a good candidate for aducanumab, provide guidance for optimal timing of MRI to detect ARIA, and discuss ARIA management strategies when the MRI changes of ARIA are detected (17). As real-world experience is gained with aducanumab and other mAbs, use recommendations will be adjusted to ensure patient safety and optimize the opportunity for efficacy.

### Monoclonal Antibodies as a Class

Monoclonal antibodies directed at amyloid plaques are the first agents to show an effect on the underlying biology of AD and to qualify as disease-modifying therapies. These agents have succeeded where many non-plaque-directed anti-amyloid therapies have failed (11). They present new requirements for patient diagnosis and monitoring including diagnostic biomarkers, genotyping, and safety assessments. They represent a breakthrough class of drugs that can initiate a new era in AD therapeutics. We are at the beginning of this era and only one agent (aducanumab) has completed Phase 3 trials. There are promising data emerging from trials of plaque-lowing mAbs including donanemab, lecanemab, and gantenerumab (13, 14, 18). These agents have different delivery approaches, dosing strategies, titration schedules, and target epitopes. Preliminary observations suggest that they may have different rates of ARIA, and comparative efficacy is unknown. It is premature to suggest that a CED will be required for all plaque lowering mAbs.

Not all anti-amyloid mAbs are plaque-lowering. Solanezumab is directed at the monomeric form of Aß. It does not lower plaque burden. A Phase 3 trial in mild AD dementia did not meet its primary outcome; it is uncertain if limited efficacy was shown (19). This mAb is very different than plaque-lowering mAbs and approaching anti-amyloid mAbs as a group does not recognize this diversity.

### Effect on Innovation

Drug development for AD is a costly and lengthy enterprise requiring extensive financial and time investment (Figure 1). Pharmaceutical and biotechnology companies must realize a return on investment to warrant committing resources to a therapeutic area. Delaying a return on mAb development costs while a CED is conducted will disincentivize drug development for AD. The balance between encouraging innovation with financial incentives while not unduly escalating drug prices is difficult to achieve and is a topic of national and international dialogue (20). Finding the right balance between innovation and drug costs will be a catalyst to developing treatments urgently needed for patients with AD. Reimbursement models such as that developed for aducanumab by the Institute for Clinical and Economic Review (ICER) --- on which CMS depended in part for the proposed coverage decision --- have no terms in their formulae for innovation and adopt primarily a health care systems perspective (21). These models provide insights but are not balanced multi-stakeholder views.

## Summary and Call to Action

CMS concludes the proposed decision memo by stating that the proposal strikes an appropriate balance of providing patient access while also ensuring both protections for patients from harms and the appropriate data collection and analysis to determine whether CMS should undertake an NCD reconsideration (3). The recommended decision does not strike an appropriate balance. Evidence of efficacy is discounted; evidence of harm is exaggerated; the purpose of accelerated approval to make drugs available while data are generated is ignored; the requirement for confirmatory trials funded by NIH and conducted in hospital settings creates undue delay; the discussion of primary trial outcomes and minimal important differences is premature and the data are extrapolated from non-trial populations; the desire for proportional representation of underserved minorities in trial populations is laudable but impractical as proposed and will increase treatment inequities; the availability of aducanumab to those who can pay for it and accessible only through trials for those who cannot afford it is improper; application of the CED requirement to all anti-amyloid mAbs is inappropriate and will stifle innovation. These issues should be addressed in a revised proposal. Alternative approaches such as registries, collection of real world evidence, examination of claims data, and post-marketing studies should be entertained. The Proposed Decision Memo is open to public comment (closing 30 days from January 11, 2022). Motivated individuals can submit comments on the CMS. gov website (https://www.cms.gov/medicare-coverage-database/search.aspx).
